# Flexible Enzymatic Glucose Electrochemical Sensor Based on Polystyrene-Gold Electrodes

**DOI:** 10.3390/mi12070805

**Published:** 2021-07-07

**Authors:** Annika Müsse, Francesco La Malfa, Virgilio Brunetti, Francesco Rizzi, Massimo De Vittorio

**Affiliations:** 1 Center for Biomolecular Nanotechnologies, Istituto Italiano di Tecnologia, Via Eugenio Barsanti 14, 73010 Lecce, Italy; francesco.lamalfa@iit.it (F.L.M.); virgilio.brunetti@iit.it (V.B.); massimo.devittorio@iit.it (M.D.V.); 2 Dipartimento di Scienze e Tecnologie Biologiche e Ambientali, Università del Salento, Via per Monteroni, 73100 Lecce, Italy; 3 Faculty of Technology and Bionics, Rhine-Waal University of Applied Sciences, Marie-Curie-Straße 1, 47533 Kleve, Germany; 4 Dipartimento di Ingegneria dell’ Innovazione, Università del Salento, Via per Monteroni, 73100 Lecce, Italy

**Keywords:** glucose, glucose oxidase, amperometric biosensor, body fluids, sweat, wearable sensor

## Abstract

Metabolic disorders such as the highly prevalent disease diabetes require constant monitoring. The health status of patients is linked to glucose levels in blood, which are typically measured invasively, but can also be correlated to other body fluids such as sweat. Aiming at a reliable glucose biosensor, an enzymatic sensing layer was fabricated on flexible polystyrene foil, for which a versatile nanoimprinting process for microfluidics was presented. For the sensing layer, a gold electrode was modified with a cysteine layer and glutaraldehyde cross-linker for enzyme conformal immobilization. Chronoamperometric measurements were conducted in PBS buffered glucose solution at two potentials (0.65 V and 0.7 V) and demonstrated a linear range between 0.025 mM to 2mM and an operational range of 0.025 mM to 25 mM. The sensitivity was calculated as 1.76µA/mM/cm^2^ and the limit of detection (LOD) was calculated as 0.055 mM at 0.7 V. An apparent Michaelis–Menten constant of 3.34 mM (0.7 V) and 0.445 mM (0.65 V) was computed. The wide operational range allows the application for point-of-care testing for a variety of body fluids. Yet, the linear range and low LOD make this biosensor especially suitable for non-invasive sweat sensing wearables.

## 1. Introduction

To prevent and optimally treat diseases, monitoring the health status of patients is of great importance. Among one of the most widespread diseases is diabetes, a metabolic disorder that affects more than 450 million people worldwide, which is characterized by persistent high blood glucose levels that cause vascular damage and affect the heart, eyes, kidneys and nerves. The number of affected people is expected to increase up to 700 million in 2045, and it is estimated that half of all people with diabetes are undiagnosed, which illustrates the large demand for glucose monitoring [1].

Typically, in a clinical setting, glucose levels are measured invasively using frequent blood sampling, either analyzed in central laboratories or directly at the patient’s bedside, known as point-of-care testing. The great advantage of using point-of-care instruments in diabetic patients is that the turn-around time is much shorter, which is crucial as changes in the blood sugar level can require immediate adjustment. In addition, less blood volume is needed, which reduces the probability for anemia due to frequent sampling [2]. In a home setting, most commonly, finger-prick testing is used, which can be painful and does not allow for frequent measurements. These drawbacks of conventional invasive glucose testing have been addressed in recent years, and increasing efforts have been made to develop minimally- and non-invasive methods [3]. Especially, wearable devices have been in focus, as they can allow for continuous measurements [4,5]. This is possible thanks to the correlation between blood glucose levels and the level in other body fluids, such as interstitial fluid, sweat and saliva (Table 1) [6,7,8].

To date, a completely non-invasive glucose sensor still is, to the best of our knowledge, not widely available for commercial use despite the high attention this technology gained recently [3,10]. The availability of large amounts of fluid that do not require withdrawal techniques makes saliva an interesting target for glucose sensing and led to the development of such sensors [11]. Yet, despite the correlation between glucose levels in blood and saliva, measurements are only reliable under fasting conditions, which limits usability for patients with diabetes and thus commercialization [12]. On a commercial level, electrochemical biosensors to measure glucose levels in interstitial fluid have been developed [13,14]. A possibility to access interstitial fluid is to access the fluid internally using needles, which penetrate through the skin. These indwelling sensors are widely available and have gained growing market acceptance [12], but potential drawbacks such as the risk for microbial infection remain [15,16]. One such commercially available sensor is the FreeStyle Libre Flash Glucose Monitoring System (Abbott Diabetes Care) with a sensor needle length of 8.5 mm [17]. In addition, less invasive sensors using microneedles to access the fluid, such as the device from Arkal Medical, have been developed [18], but swelling and irritation can occur each time the sensor breaches the skin [12]. Withdrawing the fluid from the skin is an alternative approach that has been exploited in laboratory [19] and by the commercially approved GlucoWatch^®^ system (Cygnus Inc., Redwood City, CA, USA) in the early 2000s [20], but the device had to be retracted from the market. Among the reasons was skin irritation due to the current that was necessary during the reverse iontophoresis process to induce fluid migration across the skin [3]. Other drawbacks of interstitial fluid sensing requiring extraction are the increased lag time and possible contamination with sweat [12].

Due to the possibility to access sweat non-invasively, this body fluid has been brought into focus, in recent years, as a good candidate to allow for non-invasive sensing, but it has been pointed out that, compared with the development of glucose sensors for interstitial fluid, the commercialization of sweat glucose sensors is still low [12]. Although sweat is easily accessible because the whole body is covered with sweat glands, high sweat rates are typically found only in people such as athletes or workers and, indeed, many sensors focus on people during physical activity to guarantee sufficient sample volumes [21,22,23]. To enable sweat sensing in resting people, often, induced sweating is employed, for example, by local application of sweat stimulants such as Carbachol or Pilocarpine using a reverse iontophoresis approach [24,25]. Further efforts have been made to develop sensors able to operate at low volume levels of 1–5 µL [26].

However, comparatively low glucose concentrations and low excretion rates in resting people represent challenges that have to be addressed by fabricating sensors with low detection limits and taking into account the suitability of microfluidic systems to collect such small volumes [27]. This means that, in order to be able to have a versatile substrate applicable also for low volume microfluidics, the material should show low absorption and low water vapor permeability, which could change the concentrations. Thus, biocompatible polymers with the aforementioned properties such as polystyrene (PS) are preferable over the widely used silicone-based elastomer polydimethylsiloxane (PDMS) to overcome some of these drawbacks [28]. In addition, faster fabrication processes are possible using thermoplastic polymers as PS [29,30].

Ever since the first electrochemical glucose sensor for blood was developed in 1962 by Clark and Lyons [31], an electrochemical approach is still being chosen for most glucose sensors. Enzyme-based sensors allow for high sensitivity and good reproducibility while production is usually possible in a low-cost range [9]. Different sensor generations are distinguished in literature based on the enzymatic reaction side [32,33]. Glucose oxidase (GOx) sensors are based on the enzymatically catalyzed oxidation of glucose to gluconolactone in the presence of oxygen. The coenzyme flavin adenine dinucleotide (FAD) is required as electron acceptor in this reaction and is then regenerated by reacting with O_2_ to generate hydrogen peroxide (H_2_O_2_) [34].

GOx(FAD) + Glucose → GOx(FADH_2_) + Gluconolactone (1)

GOx(FADH_2_) + O_2_ → GOx(FAD) + H_2_O_2_ (2)

When a sufficient potential is applied at the electrodes, H_2_O_2_ is oxidized, and a current can be measured which correlates with the amount of H_2_O_2_ that has been produced and thus correlates indirectly with the glucose concentration present in the fluid.

H_2_O_2_ → O_2_ + 2H^+^ + 2e^-^ (3)

The aim of the study was to obtain a simple enzymatic glucose sensor with a range suitable for sweat glucose sensing (see Table 1) to be integrated in a microfluidics in order to obtain a wearable device with an efficient sweat collection. The sweat sensors analyzed in Table 2 usually show the sweat collecting system being fabricated on top of the functionalized electrode. Here, a fabrication process for microfluidic systems was adapted to suit a PS nanoimprinting process that allows for a versatile, scalable and cost-effective fabrication in the context for wearables as well as point-of-care testing. A combination of a nanoimprinting lithography of a microfluidic on a PS substrate followed by electrode definition by metal evaporation enables a route for a mass production of wearable sweat sensors preserving the mechanical and electrical integrity of the electrodes. This approach can be worthwhile for envisioning a fast roll-to-roll production of non-invasive wearable sensing systems for sweat and other biological fluids.

## 2. Materials and Methods

### 2.1. Materials and Reagents

For the electrode fabrication, polystyrene (PS) foil with a thickness of 0.19 mm was purchased from GoodFellow (Prodotti, Gianni S.r.l., Milan, Italy) and adhesive foil sheets were obtained from Greiner Bio-one (platesealer EASYseal^TM^ transparent, RS Components S.r.l., Milan, Italy). Isopropyl alcohol (IPA), L-cysteine (BioUltra, ≥98.5% (RT)), phosphate buffered saline tablets (PBS), Bovine serum albumin (BSA, lyophilized powder, ≥96%), glycerol (≥99%), glutaraldehyde (GTA, Grade I, 70% in H_2_O), glucose oxidase (GOx, from *Aspergillus niger*, Type X-S, lyophilized powder, 100,000–250,000 units/g), D-(+)-Glucose (≥99.5%) and potassium hexacyanoferrate(II) trihydrate (98.5–102.0%), were provided by Sigma Aldrich (Merck Life Science S.r.l, Milan, Italy). Deionized (DI) water was taken from a Milli-Q^®^ water system (Millipore).

For fabrication of the microfluidics, the following material was used: Si wafer, polyester film photomasks (JD Photo Data, Hitchin, UK), SU-8 2002 and SU-8 2100 photoresist, SU-8 developer (MicroChem Corp, Newton, MA, USA), UV-glue (NOA 68, Norland Products Inc, Cranbury, NJ, USA).

### 2.2. Electrode Fabrication

For the experiments to characterize the properties of the working electrodes (WE), these were fabricated as single electrodes on PS foil, and measurements were taken in a electrochemical cell with a separate silver/silver-chloride (Ag/AgCl) reference electrode and a separate platinum (Pt) counter electrode; whereas for the first experiments for integration with microfluidics, the WE was combined with a counter electrode (CE) and a reference electrode (RE) on the same PS foil substrate. In all three electrodes, a 5 × 8 mm contact pad and a 10 × 0.5 mm wire connection were present, leading to the electrode surface in contact with the fluid. The circular shaped WE had a diameter of 4 mm, and, in the three-electrode system on PS foil, it was centered between the CE and RE; both CE and RE were bent in half-circular shape surrounding the WE in order to allow for the CE and RE to be close to the WE, exploiting a large surface area. The PS foil was cleaned using IPA and DI water and then dried using nitrogen flux. Using a laser cutter (VLS2.30DT, Universal Laser Systems GmbH, Wien, Austria), the design was cut into adhesive foil sheets that were attached on the PS foil to serve as mask in the following metal evaporation process. First, a thin adhesion layer of chromium (about 15 nm) was thermally evaporated on the PS film followed by a gold (Au) layer (about 150 nm). Then, the adhesive mask was carefully removed and excess PS film was cut. For the RE, Ag (about 100 nm) was thermally evaporated using a new mask. The maximum deposition rates were up to 1 Å/s.

### 2.3. Electrode Functionalization with GOx

Prior to the functionalization of the WE electrode, the samples were washed with IPA and DI water. Then, the electrode was covered with 50 mM L-cysteine solution for 20 h at room temperature (RT) to create thiol–gold bonds, followed by washing with DI water and BSA solution (30 mg/mL BSA in PBS). After that, 30 µL drops of GTA solution (2.5 wt% GTA, 50 mg/mL BSA and 1 vol% glycerol) were applied on the electrode surface for 2–3 h at RT to immobilize GOx via cross-linking. BSA and glycerol contributed to the stabilization. The electrodes were then washed with BSA solution to which glycerol was added (30 mg/mL BSA and 1 vol% glycerol in PBS). Next, 30 µL drops of GOx solution (25 mg/mL GOx in PBS) were placed on the electrode for 2.5 h at 4 °C. Finally, electrodes were washed with PBS solution and stored at 4 °C in PBS.

### 2.4. Electrode Characterization

For the electrochemical characterization of the WE electrode, experiments were conducted in an electrochemical cell with external RE (Ag/AgCl in KCl) and external CE (Pt sheet) using a potentiostat (Autolab, Metrohm Autolab, The Netherlands) and the software NOVA (Metrohm Autolab). Cyclic voltammetry (CV) was performed on Au electrodes without functionalization from −0.2 V to 0.6 V at different scan rates (10, 20, 40, 50, 60, 80, 100, 140, 180, 200 mV/s) in [Fe(CN)6]^3−/4−^ (ferro-ferricyanide) solution for confirmation of the response of the electrode. Further, Au electrodes were tested for their current response in different concentrations of H_2_O_2_ (0–25 mM) at 0.7 V. Chronoamperometry (CA) was performed by placing the GOx-functionalized WE, the Ag/AgCl RE and the Pt CE in the electrochemical cell filled with 20 mL of PBS. After a stable baseline current was reached, glucose stock solution (2 M in PBS, prepared the previous day to allow for mutarotation of the glucose) was added stepwise to the PBS solution until a maximum glucose concentration of 25 mM was reached. The applied potentials were 0.65 V and 0.7 V at a constant pH of 7.4, which is the standard physiological buffer. To test for a possible influence of the pH, the current response was measured for pH values between pH 4.5 to pH 8 in glucose solution (pH adjusted in 1 mM glucose in PBS) and at potentials ranging from 0.5 V to 0.75 V. Sensitivity was calculated as the slope for the linear range divided by the circular electrode area, and the limit of detection (LOD) was calculated as three times the standard deviation of the baseline current divided by the slope [37]. The apparent Michealis–Menten constant Km(app) was calculated using the software OriginPro 2018 (OriginLAB, USA) following the Lineweaver–Burk formula:

1/I_SS_ = 1/I_max_ + (Km(app))/I_max_) × 1/c, (4)

where c represents glucose concentration, I_SS_ is the steady-state current at a certain glucose concentration and I_max_ describes the maximum current under saturated conditions.

### 2.5. Microfluidics Fabrication for Further Device Integration

A nanoimprinting approach was used to fabricate microfluidics in PS in order to be integrated with the electrodes. Photomasks for the fabrication of a SU-8 stamp were designed using the software CleWin (WieWeb software, Hengelo, The Netherlands) and printed on photomask foil. To fabricate the stamp, a photolithography process was exploited: After oxygen plasma treatment for surface activation (100 W for 5 min), a 2 µm layer of SU-8 2002 photoresist was spin coated on a clean 2” Si-wafer substrate after the following protocol: 500 rpm for 5 s, then 1800 rpm for 30 s; followed by a soft bake at 95 °C for 1 min 30 s; cool down period of 10 min; flood exposure under UV light (1 min 30 s at about 6 mW/cm^2^ at 365 nm wavelength); post-exposure bake at 95 °C for 1 min 30 s; development for 1 min. The purpose of this thin SU-8 layer was to improve the attachment of the following thicker SU-8 layer during the imprinting process. In a second step, a layer of 110 µm of SU-8 2100 was spin coated: 1200 rpm for 90 s; soft bake for 5 min at 65 °C, then 45 min at 95 °C; UV exposition with 250 mJ/cm^2^ using the photomask; post-exposure bake for 5 min at 65 °C, then 30 min at 95 °C; development 5 min; hard bake for 10 min at 150 °C. The obtained SU-8 structure is the negative of the desired microfluidic pattern to be transferred to the polymer substrate. The PS foil was rinsed with DI water and dried using nitrogen flux prior to the nanoimprinting process. The SU-8 stamp and the PS foil were stacked, and the pattern was imprinted at a temperature of 140 °C for 300 s using the nanoimprinting instrument (EITRE 3, Obducat). The channel height was measured using a profilometer (Bruker Dektat XT) resulting in a final value of 105 µm. At this stage, the electrode definition is conducted by metal evaporation with the same procedure as described in Section 2.2. The PS sample was then exposed to oxygen plasma at 200 W for 10 min (RFG 300, Diener) to hydrophilize the surface. A closed microfluidic system was obtained by applying UV glue at the borders of the microfluidics and irradiated for 1 to 1 min 30 s.

## 3. Results

### 3.1. Electrochemical Characterization

**Figure 1:**
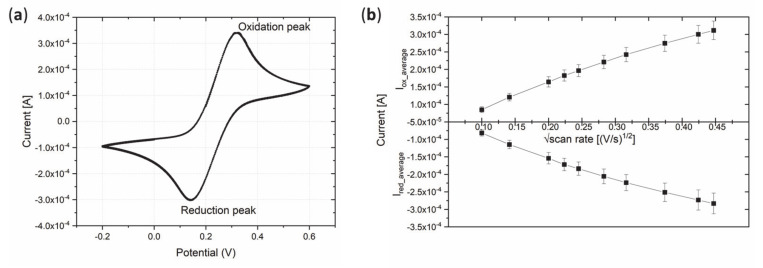
Cyclic voltammetry of a Au electrode on polystyrene (PS) foil in ferro-ferricyanide solution. (**a**) Exemplary cyclic voltammogram. (**b**) Square root of the scan rate vs. maximum absolute values of reduction and oxidation currents (*n* = 6). *n* = number of analyzed samples.

CV was performed on the Au electrodes in ferro-ferricyanide solution to characterize the current response to evaluate the electrode fabrication process for its suitability and reliability. Figure 1a shows an exemplary CV with both oxidation and reduction peak demonstrating the reversibility of the redox reaction. In addition, Figure 1b shows a plot of the square root of the scan rate against the maximum oxidation and reduction currents. The second plot showed increasing absolute current values for higher scan rates, which was expected for Au electrodes in ferro-ferricyanide solution. The characterization showed a good repeatability among several samples; thus, the fabricated Au electrodes were suitable for further functionalization steps.

**Figure 2:**
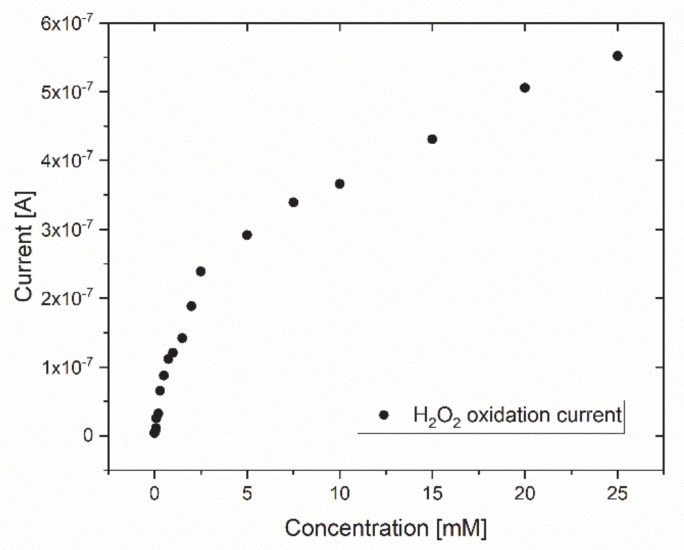
Exemplary current response of a Au electrode in different H_2_O_2_ concentrations. The trend showed an increasing current response with increasing H_2_O_2_ concentrations.

The ability of the Au electrode to detect current changes in different H_2_O_2_ concentrations was demonstrated as the measured current increased with increasing H_2_O_2_ concentrations (Figure 2). This is of importance as the sensor working principle relies on a current response to the H_2_O_2_ oxidation process that occurs as a result of the enzymatic glucose catalysis. As the chosen potential of 0.7 V vs. Ag/AgCl resulted in this reliable current response, further experiments were conducted at this potential.

**Figure 3:**
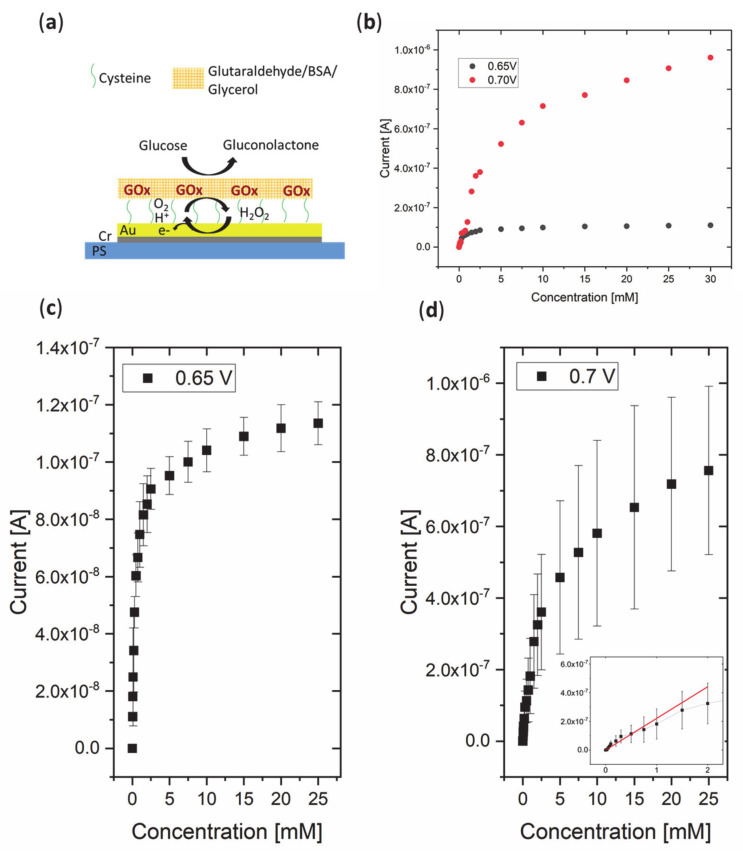
(**a**) Scheme of sensing mechanism of the glucose oxidase (GOx)-functionalized Au electrode. (**b**) Exemplary calibration plots of the GOx-functionalized Au electrode in different glucose concentrations for (**c**) 0.65 V (*n* = 3) and (**d**) 0.7 V (*n* = 3). A linear increasing current response for increasing glucose concentrations was found between 0.025 mM and 2 mM at 0.7 V (inset), whereas the operational range was up to 25 mM at both potentials before saturation occurred. *n* = number of analyzed samples.

Glucose sensing was achieved by the functionalization of the Au electrode with GOx, where thiol–gold bonds, thanks to the cysteine self-assembled monolayer (SAM layer), acted as a link between the enzyme and the Au surface, and the GTA/BSA/glycerol network enhanced immobilization and stability (Figure 3). Chronoamperometric measurements were conducted at 0.65 V and 0.7 V to demonstrate suitability for glucose sensing. In addition to an applied potential of 0.7 V, a slightly lower value of 0.65 V was chosen to test for the sensor performance, as lower potentials can be advantageous to reduce interference with other species [38]. A linear increase in the current was observed between concentrations of 0.025 mM and 2 mM at 0.7 V, whereas the whole operational range was observed to be between 0.025 mM and 25 mM of glucose for both 0.65 V and 0.7 V, as saturation occurred at concentrations higher than 25 mM (see Figure 3). Comparing the current between an applied potential of 0.65 V and 0.7 V, it is notable that the overall current is higher for 0.7 V. The sensitivity was calculated as 1.76 µA/mM/cm^2^ at 0.7 V. The LOD was calculated to be 0.055 mM at 0.7 V. The linear range obtained at a potential of 0.7 V made the higher potential seem more favorable. However, it has to be noted that, at a higher potential, the baseline current was higher and showed higher noise levels, and a 5–10 min longer period of time was required at 0.7 V potential before a stable baseline current was obtained. The apparent Michaelis–Menten constant was computed as 0.445 mM for 0.65 V and 3.34 mM for 0.7 V, which showed that GOx had a higher substrate affinity at the lower potential. However, the lower substrate affinity at 0.7 V showed better suitability for the determination of glucose concentrations typically present in sweat and other body fluids. As shown in Figure 3b, at 0.7 V the Michaelis–Menten curve shows a saturation at higher concentrations which implicates that, even at higher glucose concentrations, the enzyme activity is not limiting the reaction rate.

**Figure 4:**
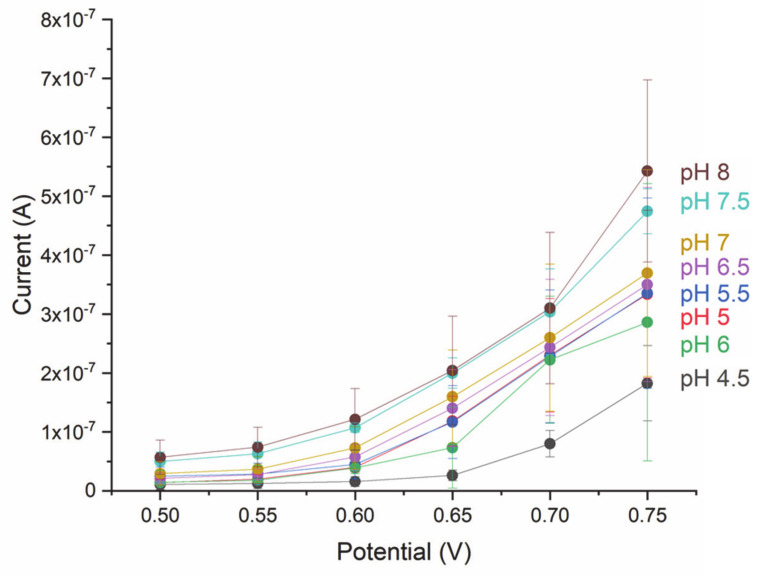
Current response of GOx functionalized Au electrode at different pH values (*n* = 3). *n* = number of analyzed samples.

To investigate the relation between the pH of the analyzed fluid and the response of the electrode, the current was measured at pH values between 4.5 and 8 for potentials between 0.5 V and 0.75 V (Figure 4). At a constant glucose concentration of 1 mM, which was chosen because this concentration is well within the linear range, the current was found to increase with increasing pH values despite the standard deviations overlapping in adjacent pH values mainly between pH 5 and pH 6. Moreover, an overall trend was found, which showed increasing current with increasing potential.

### 3.2. Microfluidics for Three-Electrode System

**Figure 5:**
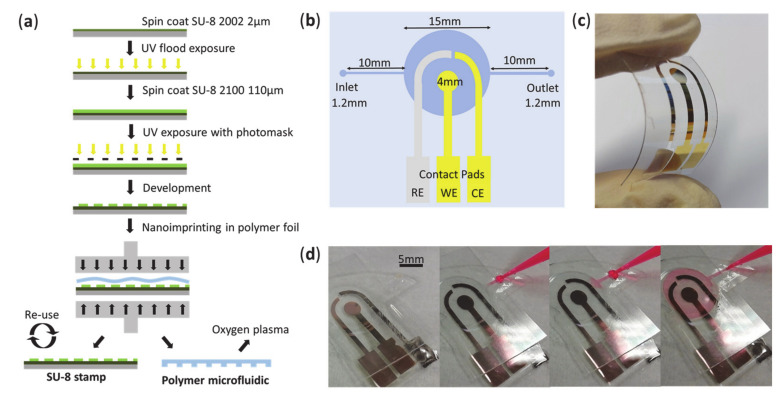
(**a**) Microfluidic fabrication process using nanoimprinting. (**b**) Scheme of the three-electrode system and a simple microfluidic channel. (**c**) Reference electrode (RE), working electrode (WE) and counter electrode (CE) evaporated on PS foil. (**d**) Application of dyed water to validate the filling of the chamber.

The nanolithography and nanoimprinting process used for the fabrication of a simple microfluidic system with inlet, outlet and a circular chamber is described in Figure 5a. Following the design scheme of the three-electrode system and the microchannel (Figure 5b), the Au WE and CE and the Ag RE were evaporated on flexible PS foil (Figure 5c) and a closed microfluidic system was tested for the fluid dispersal using dyed water, which showed uniform fluid flow over all electrodes (Figure 5d).

## 4. Discussion

A first generation amperometric biosensor for glucose was realized by immobilizing GOx on an Au electrode that was evaporated on flexible PS polymer foil. The enzyme was attached to the Au surface via thiol-bonds created by a cysteine SAM layer, and embedded into the cross-linker GTA. BSA and glycerol were added for stabilization purposes.

The response to different glucose concentrations was evaluated by chronoamperometric measurements, and a good sensor output for the biologically relevant range in body fluids such as blood, interstitial fluid, sweat and saliva was demonstrated. Comparing the sensor performance at 0.65 V and at 0.7 V showed that an LOD of 0.055 mM glucose and sensitivity of 1.76 µA/mM/cm^2^ were obtained for the higher potential, whereas, at 0.65 V, no linear range was shown. A higher substrate affinity was found at 0.65 V given by the apparent Michaelis–Menten constant of 0.445 mM compared with 3.34 mM at 0.7 V. The low Km(app) value at 0.65 V compared with a similar sensor fabricated with GTA for cross-linking (Km(app) of 1.15 mM [35]), could be due to the stabilizing effect of BSA and glycerol. In general, the obtained results for the sensor are in line with the previous work based on a GOx sensor; however, a comparatively large operational range was obtained (Table 2), also possibly due to the addition of BSA and glycerol as stabilizing agents [39]. To reduce possible interference effects in body fluids, an electrostatically charged and porous membrane such as a nafion layer could be added [40]. Further testing and assessing the optimal potential can help to find the best trade-off between the sensor characteristics depending on the requirements for the field of application.

The linear range of 0.025 mM to 2 mM and the large operational range of 0.025 mM to 25 mM make the sensor suitable for sensing glucose concentrations in a variety of body fluids (see Table 1). A possible field of application of the sensor is its use as a disposable strip for point-of-care measurements to detect the glucose concentration, which is to be used by medical professionals. In this context, the whole three-electrode system can be placed on the same strip, so the presented electrode fabrication process demonstrated the first steps for further integration. Wearable device market applications could be envisioned, especially sweat sensors that allow for non-invasive glucose monitoring.

Several adaptations should be considered for a reliable sweat sensor. In fact, it is known that sweat pH can vary between 4.5 and 7 [41], and the results showed that the current varies depending on the pH. Therefore, it could be of advantage to integrate a pH sensor to achieve a more complete calibration. In addition, it is noteworthy that a response maximum was expected at a slightly acidic pH, as the pH optimum of most GOx is between a pH of 5 and 6 [42]. The different behavior of GOx on the sensor surface could be due to nonspecific modifications of the enzyme surface during the fabrication process [43], and further work is necessary to gain full understanding. Another important adaptation regards the RE: When fabricating a reliable RE for sweat, the presence of chloride ions needs to be considered. In case of the Ag/AgCl RE, an additional layer is required to avoid sensing the presence of chloride ions. Such an electrode can be realized by chemically converting the evaporated Ag electrode into an Ag/AgCl electrode and subsequently adding a layer of polyvinyl butyral [44]. As secreted sweat volumes range between 0.1 and 2 µL/min/cm^2^ [12], the miniaturization of the electrodes will be of advantage to collect and drive such small amounts of fluid. On that account, the presented fabrication technique for the microfluidics is highly versatile and allows to easily adapt to smaller structure sizes. Even other thermoplastic materials can be used for the nanoimprinting process, among these, more flexible polymers such as soft thermoplastic elastomers and specifically styrenic block copolymers that can adapt well to the human skin because their Young’s modulus is more similar to that of skin [45]. Long-term-stability and testing of real body fluid samples will be of importance during the development of a wearable sweat sensing device that allows for continuous glucose measurements and, by this, paving the way for a new generation of non-invasive glucose sensors improving the quality of life of diabetic patients.

## Figures and Tables

**Table 1:** Typical glucose concentrations in different types of human body fluids. Data from ref. [9].

Body Fluid Type		Blood	Interstitial Fluid	Sweat	Saliva
Glucose concentration	Healthy patients	4.9–6.9 mM	3.9–6.6 mM	0.06–0.11 mM	0.23–0.38 mM
	Diabetic patients	2–40 mM	1.99–22.2 mM	0.01–1 mM	0.55–1.77 mM

**Table 2:** Comparison of present work to other amperometric glucose biosensors.

Operational Range [mM]	Limit of Detection [mM]	Sensitivity	Ref.
0.025–25	0.055	1.76 µA/mM/ cm^2^	Present work
1.5–7	0.94	2.65 µA/mM/ cm^2^	[35]
0.01–0.7	0.01	1 µA/mM	[36]
0–0.1	-	2.1 µA/mM	[25]
0–0.2	-	2.35 nA/µM	[22]
0.005–1	-	-	[11]
0.05–0. 2	-	3.29 nA/µM	[21]
2–10	0.05		[23]
0.005–2.8	0.005		[26]
0–0.1	0.003	23 nA/µM	[19]

## Data Availability

Not applicable.
